# Community Walking in People with Parkinson's Disease

**DOI:** 10.1155/2012/856237

**Published:** 2011-11-29

**Authors:** Robyn M. Lamont, Meg E. Morris, Marjorie H. Woollacott, Sandra G. Brauer

**Affiliations:** ^1^School of Health and Rehabilitation Sciences, The University of Queensland, Brisbane, QLD 4072, Australia; ^2^Melbourne School of Health Sciences, The University of Melbourne, Melbourne, VIC 3010, Australia; ^3^Department of Human Physiology, University of Oregon, Eugene, OR 97401, USA

## Abstract

People with Parkinson's disease often have walking difficulty, and this is likely to be exacerbated while walking in places in the community, where people are likely to face greater and more varied challenges. This study aims to understand the facilitators and the barriers to walking in the community perceived by people with Parkinson's disease. This qualitative study involved 5 focus groups (*n* = 34) of people with Parkinson's disease and their partners residing in metropolitan and rural regions in Queensland, Australia. Results found that people with PD reported to use internal personal strategies as facilitators to community walking, but identified primarily external factors, particularly the environmental factors as barriers. The adoption of strategies or the use of facilitators allows people with Parkinson's disease to cope so that participants often did not report disability.

## 1. Introduction

Community ambulation is compromised in many people living with Parkinson's disease (PD), which is thought to affect around 2 percent of the population over the age of 65 [1]. Gait changes are a hallmark of PD, and people with PD frequently walk with reduced speed and step length [2, 3], reduced cadence [2–5], and increased gait variability [6]. People with PD may also experience freezing when walking. Walking difficulties are exacerbated when attention is drawn away from walking by performing additional tasks [5–9]. Challenging environments that demand attention may also compromise the ability to walk in people with this debilitating condition.

Community walking is an important enabler to participation in community activities and a range of societal, work, and leisure roles. It has been defined as locomotion in environments outside the home or the residence [10]. This includes the ability to negotiate public and private venues both indoors and outdoors that incorporate a variety of environmental demands [10, 11], which could prove challenging for people with PD.

The physical, social, and attitudinal environments are generally more varied and less predictable in the community than for the home or the laboratory settings. Walking in the community is generally assumed to be a more complex and high-level skill than walking around the home or in the laboratory. Research in older adults suggests that loss of walking function is a gradual process which results in a restriction of the variety of places they go to and the distance they will venture from home [12]. Impairments can accelerate this, and disabled older adults report fewer encounters with and greater avoidance of physical challenges in the environment [13]. 

People living with PD have walking challenges in addition to the usual ageing process. The impact of these challenges on community walking is not yet understood. A greater understanding of the perceived factors (both internal and external to the person) that positively and negatively impact on the ability of people with PD to walk in the community is needed. Understanding these factors may allow clinicians to design assessment tools more appropriate for measuring community mobility deficits and provide a basis for the development of interventions to improve community mobility and potentially participation in people with PD. The aim of this qualitative study is to understand what specific facilitators and barriers individuals with PD perceive affect their ability to walk successfully in the community.

## 2. Methods

A qualitative study design was used to allow data to be gathered directly from people living with PD. Focus groups were used with the aim of encouraging discussion of a variety of experiences and opinions. Data collection ceased when saturation of the data was achieved.

### 2.1. Participants

People with PD and partners of people with PD were recruited using advertising in local PD Association publications in Queensland, Australia. Participants were eligible for the study if they or their partner had PD or they cared for someone with PD, were able to sign informed consent, and able to attend a focus group in a community setting. 

Five focus groups were conducted (*n* = 34) including three metropolitan groups of people with PD and their partners (*n* = 22), one metropolitan group of partners only (*n* = 6), and one rural group (*n* = 7). A partner group was included as it was felt that partners of people with PD could have a valuable contribution to make to this data collection but that some may be reluctant to honestly express their feelings regarding the ability of their partner if they were present. The group of partners of people with PD was purposively sampled using a database of people willing to participate in research related to PD. Demographic information about the participants is included in Table 1.

### 2.2. Procedure

Each focus group included the participants, a facilitator, and a scribe who took field notes regarding group dynamics, nonverbal communication, and interviewing conditions. Groups lasted one to two hours and were audio recorded. Prior to each focus group, participants were given written information outlining the aim of the research, the procedure for the session, and an outline of the 4 key questions (see Table 2) for discussion. They were given the opportunity to ask any questions, provided written informed consent, and completed a short questionnaire of general demographic information.

Key questions were open ended so responses were in participants' own words. Probing questions were used when needed, but every effort was made to maintain a natural discussion. At the end of each focus group, the facilitator summarized the main points of the discussion and her perceptions. Participants were asked to confirm the accuracy of this summary.

Approval for this study was obtained from the University of Queensland's Behavioural and Social Sciences Ethical Review Committee (Application #2008001843).

### 2.3. Analysis

Immediately after each group, the facilitator reflected on the discussion with the aim of putting aside any immediate thoughts or judgments so the next group was approached with minimal preconceptions.

All audio recordings were professionally transcribed verbatim by professionals external to the study. To confirm accuracy, members of the research team checked each transcription twice against the audio file. Two researchers (RL & SB) then performed thematic content analysis of the transcripts, using a process of repeated readings. Initial reading aimed to capture the context of the entire discussion. Further readings aimed to identify themes that were emerging with notes initially made in the margins identifying noteworthy phrases, lines, and paragraphs of the prose. These were analysed, asking first “what does this mean?” and then “how is this the same/different to other segments?” [14]. At this point the two researchers met to discuss the themes each had identified and classify the distinctive features of these themes. Subsequent readings of the transcripts were performed to ensure the accuracy of the themes and to identify sections of discussion consistent and inconsistent with these themes.

At this point the researchers performed an analysis of the existing literature. This ensured that themes were drawn solely from the data without influence of preconceived ideas interpreted from the literature.

## 3. Results

Eighteen people with PD with a mean age of 67 years (range 41–82 years) and mean disease duration of 10.3 years (range 4–21 years) participated in the study. Freezing was reported by 44% (8) of participants, and 33% (6) reported falls in the prior 6 months (Table 1). Twenty-two partners who had a mean age of 65.4 (range 39–78) were also included. 

Three primary themes emerged from the data: (i) people with PD used internal and external facilitators to make walking in the community easier, (ii) they perceived barriers to be primarily external environmental factors, and (iii) due to their effective use to/of facilitatory strategies, many people with PD did not report community walking disability. These will be outlined in turn.

### 3.1. Facilitators

Several factors which contribute to the ability of a person to walk in the community were discussed by the groups. These are termed facilitators and included both internal factors driven by the person and external factors mediated by objects or people outside the person with PD. Internal factors were often strategies people adopted to ensure they could continue to optimally walk in the community. These could be spontaneous strategies, used to cope with a particular situation or symptom as it arose, planned in advance to maximise the chance of success, or may have become a normal behaviour now used without compromise.

#### 3.1.1. Internal Facilitators

A common strategy described was consciously attending to walking speed, step length, and toe clearance. This strategy was reported to be used to respond to challenges to walking when they arose. Most people who reported gait changes described using this strategy as either concentrating on their walking or taking extra care with walking.

“But if you walk slower and lift your feet and concentrate that helps” (PD-27).

While thinking of taking long, rhythmical steps was commonly used to aid walking in the community, it was reported that remembering to use this strategy in a community environment may be less automatic than when at home.

“…you've got to try and think and remember to do it, like, think and make sure you do it … try and step it out and lift your feet more” (PD-27).

Planning and preparation played a role to ensure walking in the community was successful. Almost everyone reported timing outings to coincide with times of high medication effectiveness (“ON” times). Being prepared for outings, making a plan and keeping to that plan reduced the chance of running late, feeling rushed, and making errors such as forgetting to take medications, and thereby reduced stress. Errands were also carefully organised to ensure the shortest walking distance.

Community walking facilitated by a novel or enjoyable situation was discussed by a number of people with PD and supported by their partners. Specifically, participants described reduced symptoms and less fatigue while travelling on holiday than they generally experienced at home, a change which could last for a number of weeks after their return.

“Going back three years when (my wife) I'd say had full blown Parkinson's, she was very, very bad. We took an overseas trip and … (my wife) just kept going and going. By the time we got to France I flaked… She still kept going… Something kept her going because as soon as we got home, boom, she got Parkinson's again, but while we were away it didn't seem to affect her” (Pa-15).

Optimising pharmaceutical or surgical interventions was a strong facilitator for some people. Optimal medication regimes were related to a more efficient gait pattern and less fatigue making long-distance walking more feasible. A positive response to surgical intervention had allowed one participant “freedom” from a schedule of medication allowing community outings to occur at times convenient for reasons other than medication effectiveness.

“I love it, I love the independence and I love being able to go to the shops and not be dictated by the medication” (PD-14).

#### 3.1.2. External Facilitators

People with PD and their partners reported that partners supported walking in the community by encouraging their partners to go out, by promoting the importance of continuing to walk as able, by providing physical assistance to overcome barriers in the environment, and by supporting the use of attention or cueing strategies. To be effective, cuing strategies needed to be discrete, mutually agreed on, and practiced to avoid using a counterproductive cue. 

Using equipment was discussed by only a few participants but included changing to more appropriate footwear and carrying a wheelchair in the car in case a long walking distance or an ineffective dose of medication was encountered. 

Only one aspect of the physical environment was described as a facilitator to community walking, but this was reinforced by many participants. Signalled pedestrian crossings reduce attention required to monitor traffic and decide when to safely cross and were thereby reported to facilitate walking in the community. For a number of participants, this had become a habit, now done without compromise.

“… you never try to run a light, you always wait for the lights and you don't cross any road if there is not a light” (Pa-11).

### 3.2. Barriers

Barriers is the term used to describe factors reported to exacerbate the negative features of their gait such as slow walking speed and, therefore, negatively influence the experience of walking in the community or cause participants to avoid walking in the community. External environmental factors were more frequently perceived to limit community walking than internal personal factors.

#### 3.2.1. External Barriers

Crowded environments were overwhelmingly disliked by most people in four of the focus groups. The exception was the rural group in which only one participant reported any particular difficulty in crowds. Participants described the need to change direction and avoid obstacles when walking in cluttered (e.g., restaurant) or heavily populated environments (e.g., shopping malls) as a trigger for short shuffling steps and more frequent episodes of freezing. Environments that are busy with people, whose actions are unpredictable, were the most frequently reported barrier.

“I find it more difficult when there are a lot of people around, it means you have to take shorter steps, I like taking long steps, I can balance myself better” (PD-6).

Attention-demanding environments such as unfamiliar environments and road crossing were not reported to contribute to any specific gait difficulty, but many participants reported a need to take extra care while walking in such environments. Road crossing was a particular problem for the rural group, which was conducted in a town that had no signalled and very few designated crossings which were inconveniently located forcing people to cross a busy highway without designated pedestrian crossings.

“Just watching for the traffic—you might not be walking as quick as you should be and you're watching for the traffic. You have to be pretty careful here” (PD-19).

Characteristics of the walking surface such as uneven footpaths, hills, ramps, flat and inclined moving walkways (travelators), and slippery surfaces were reported as a cause of increased fatigue (hills), fear of falling (uneven and slippery surfaces), and more frequent freezing episodes (ramps and travelators). Even the camber of the footpath, designed to allow water to drain, was commonly reported to make walking more difficult.

“My greatest difficulty when I'm walking is going downhill—can't handle it, I can go uphill flat out, but I can't handle going downhill. Even with a trolley my feet get stuck on top of a ramp and I can't get going” (PD-2).

The rural group specifically emphasised this barrier. In this rural town, footpaths are often absent, where present some of the footpaths are tiled and slippery when wet, and the gutters very deep (20–25 cm high) making access from the road to the footpath difficult.

Inclement weather and reduced or fluctuating lighting were reported to increase difficulty of walking and fear of falling. For some participants these were reasons to avoid community walking all together.

“We avoid going out when it's raining. It makes him want to walk faster and he gets so fast that he shuffles” (Pa-10).

#### 3.2.2. Speed Demands

Only a small number of participants reported difficulty walking as fast as the environment demanded. This was often associated with an inability to walk quickly enough to cross the road. One partner reported that his wife felt unable to walk quickly enough for him to achieve exercise benefits so she no longer walked with him for exercise.

“I'm not a quick walker, but it's quicker than she is and I don't mind walking slower but she feels she is holding me back … that I'm not getting the exercise” (Pa-33).

Walking distance was described as a barrier only in the rural group. Often these participants related greater walking distance to greater fatigue and avoided walking in the community if long distances were encountered.

“because (my partner) can't walk or stand for a long time, if we can't get a park close to somewhere where we want to go we just come home” (Pa-18).

#### 3.2.3. Internal Barriers

Participants reported that their response to PD medication was unpredictable and walking when medication was not effective very difficult. For some participants this meant that trips needed to be postponed, modified, or abandoned due to an ineffective dose.

“I'll say, right, we're going down to the shops in half an hour—take medication, might get to the shops, medication doesn't work—(we have to) come home” (Pa-13).

Even with predictable “ON” and “OFF” times, one participant with PD reported that her need to schedule outings for times that medication would be effective gave her a feeling of being “locked to the medication” (PD-14). This on-off phenomenon was also reported as one source of anxiety.

“What if I get weak, what if I can't move, what if I've got to come home straight away?” (Pa-13).

Anxiety was reported to increase symptoms of PD, resulting in walking difficulty such as shortened step length and increased “shuffling” or dragging a leg. Feeling hurried, examined, stigmatised, or judged was also reported to increase anxiety.

“… walking down here this morning I thought I would be late and I started dragging my foot again” (PD-19).

Some participants reported fatigue due to longer than usual walking distance or time. As a result of fatigue, people reported abandoning some outings before they had intended or experiencing fatigue-related weakness and a resultant increase in walking difficulty.

“You get a fatigue coming in. You will notice it in a weaker muscle group—you might pick it up in the calf where you use it a lot. You might pick it up a hamstring or the front of the leg where it just becomes harder” (PD-17).

### 3.3. Disease without Disability

The final theme that emerged is that while strategies and facilitators are effective at overcoming barriers to community walking, people living with PD may not appreciate or report any actual problems or difficulty but rather modifications they have made to their walking. This suggests that despite the presence of disease and impairment some people with PD are able to use facilitators and strategies to overcome barriers to community walking so effectively that no difficulty or disability is consciously appreciated, even by their partners.

“I find it is not difficult, you just have to be careful in shopping centres with people left right and centre and you have to keep on the straight and narrow and put your foot in the right place” (PD-2).

“You haven't had a problem really, have you? You just have to think about it” (Pa-22).

It is clear, however, from the barriers outlined above that some people with PD are aware of difficulties they face walking in the community, and some reported very significant walking disability.

“I don't go out on my own (anymore), I have a carer who takes me out” (PD-27).

Which indicates that for some people with PD barriers become too significant to overcome using strategies and facilitators, and disability becomes appreciable.

## 4. Discussion

Walking has been reported to be the first activity of daily living that people with PD identify as having difficulty with, followed closely by a number of activities dependent on walking such as travelling and shopping [15]. To our knowledge this is the first paper published with such a broad focus, where the term community walking is used to capture walking in the community for all reasons including but not exclusive to exercise or physical activity, activities of daily living, and leisure activities. Research in other populations has investigated personal and environmental barriers and facilitators to physical activity [16]. 

The results demonstrate that people living with PD appreciate that the ability to walk in the community is the result of a successful interaction between themselves, including their disease and associated impairments, and the environment (physical and social) in which they walk. Factors reported to negatively influence this relationship were primarily dimensions of the physical environment which previous authors have labelled density (crowding and clutter in the environment), attention, terrain, ambience (weather and lighting), and temporal demands [10]. Not only do these dimensions present challenges for people with walking impairment, but for people living with PD certain dimensions can exacerbate the negative features of gait. For example, having to stop walking and change direction while walking in crowded environments demands frequent stopping, starting, and changing direction, thereby, not allowing people to walk at their preferred speed. This dimension may be particularly challenging for people who experience freezing of gait as turning and negotiating obstacles are known triggers for freezing [17]. In addition, monitoring the environment for obstacles while walking may divert attention away from walking, something that laboratory testing has demonstrated people living with PD have particular difficulty with [5–9].

The results also suggest that the interaction is further complicated for people living with PD whose impairments are not static but may fluctuate significantly depending on the effect of their medication, anxiety, and fatigue. In one qualitative study of fatigue in people living with PD, all participants agreed that fatigue had a significant and deleterious effect on their daily activities, social and leisure time [18]. Two types of fatigue are problematic for people with PD, peripheral and central fatigue [19]. Peripheral fatigue was discussed here as fatigue related to increased walking distances, and muscle fatigue related to overuse. Central fatigue is poorly understood and not discussed among any of these groups. Possibly people who suffer from central fatigue are less inclined to commit to outings and were, therefore, inadvertently excluded from this study. This may also be true of depression, which was also not mentioned in any of these groups.

This sample also reported factors that facilitated walking in the community. Primarily these facilitators were internal to the person and involved modifying their behaviour or using strategies to overcome barriers and exploit extrinsic facilitators so they may continue to walk in the community. For many this behaviour modification is so successful that, despite the presence of disease, disability or difficulty is not perceived. This phase between disease and disability may be consistent with the phase of preclinical disability experienced during aging [20]. In older adults, preclinical disability is characterised by reports of no difficulty performing a particular task, but rather reports of modification in the method or frequency of performing that task [20]. People who reported having modified how or how often they walked half a mile or climbed ten steps were found to be 3-4 times more likely to develop disability in the subsequent eighteen months [21]. 

The current study of walking in community environments adds to a recent qualitative study by Jones et al. [22] which focused on understanding challenges and strategies for everyday walking in people with PD. Jones et al. asked people with PD to reflect on the challenges and strategies they used to address the challenges to walking, both indoors and out. Walking whilst doing something else and walking in different environments were two factors identified to increase the challenge of walking. Specifically, participants strongly disliked walking in busy and crowded environments. Participants in that study also described two attention-based strategies that their sample described using to improve their walking; these were consciously monitoring their walking performance and directing attention to correct their gait pattern. 

Although some findings are similar, this study differed to the Jones et al. study in a number of ways. The focus of this study was specifically community walking, and as such the community-specific barriers and facilitators are presented in much greater detail, particularly the environmental barriers. Data was collected using focus groups, rather than in-depth interviews which may have yielded greater reflection on the topic, particularly by those participants who reported modifications to their walking without appreciable difficulty or disability. The participants of partners in the groups may have also contributed to this reflection. Finally this study was a stand-alone qualitative study with broad inclusion criteria. As such, people with unstable and unpredictable “on” and “off” phenomenon, dyskinesias, and significant walking disability were included. Participants were on average 10 years after diagnosis with 44% reporting freezing and one-third falling in the past 6 months. The findings reflect the attitudes of those who currently access the community, and as such the results may not be generalisable to all people with PD in all stages of the disease process. 

Assessment of community walking has previously been inferred by assessing an individual's gait speed and endurance in an uncluttered environment [11]. The findings of this study suggest that assessment tasks that incorporate potentially challenging environmental dimensions such as density, attention demands, terrain characteristics, or ambience could provide more specific information about the particular demands for an individual and how they modify their gait to cope. Self-report tools such as the ambulatory self-confidence questionnaire (ASCQ) [23] and the environmental analysis of mobility questionnaire (EAMQ) [24] do address some of these issues; however, their accuracy and utility in the PD population is yet to be examined. Furthermore, self-report and actual ability may not always correlate. Mobility test batteries have been developed to reflect some demands of community mobility [25] but may not include situations that people with PD find challenging or can include tasks that may be inappropriate.

Wearable sensors such as pedometers, gyroscopes, and accelerometers have been used to demonstrate changes in activity in people with PD [26–28]. These and other technologies have the potential to be developed to measure people with PD walking in challenging environments and to possibly monitor their performance when walking in the community. 

Therapeutic intervention to manage, prevent, or delay community walking disability is equally complex. The results of this study suggest that for people with PD the primary barriers are external environmental factors. Although advocacy for modifying or planning environments that would be more easily negotiated by people with PD may go some way to improve the ability of people with PD to walk in the community; environmental modification may be less feasible in the community than in a home environment. As such, a more individualised approach to intervention may focus on enhancing likely personal facilitators. This could include educating about barriers, facilitators and sharing successful strategies used by others, in addition to promoting the use of internal strategies such as attention to walking speed and step length, and planning for outings. Evidence for the use of interventions to improve community mobility in people with PD is needed.

## 5. Conclusion

This study reports the perspectives of people with PD and highlights the effectiveness of personal strategies and facilitators to enable people with PD to continue walking in the community. People with PD often find environmental challenges barriers to walking in the community but do not tend to report disability; rather, they modify their behaviour. Current clinical methods of assessing community mobility which focus on gait speed or distance, thus, may not provide sufficient information to accurately reflect a person's ability to walk in the community. Furthermore, a deeper understanding of preclinical walking disability, in people with PD, may allow therapists to provide more timely assessment and therapy, thereby, delaying the onset of disability rather than attempting to reverse disability after it presents.

## Figures and Tables

**Table 1:** Demographic information of study participants.

Participant number	PD/partner	Age (yrs)	Disease duration (yrs)	Freezing of gait	Falls in past 6 mths	Group type
1	PD	62	12	No	5	Metro
2	PD	75	21	Yes	16	Metro
3	PD	64	12	No	0	Metro
4	PD	82	5	No	0	Metro
5	PD	65	6	No	10	Metro
6	PD	71	19	No	Daily	Metro
7	PD	58	15	No	0	Metro
8	PD	78	7	Yes	6	Metro
9	PD	78	15	No	0	Metro
10	PA	—	—	—	—	Metro
11	PA	—	—	—	—	Metro
12	PD	63	11	Yes	5	Metro
13	PA	57	—	—	—	Metro
14	PD	60	12	Yes	0	Metro
15	PA	64	—	—	—	Metro
16	PD	54	6	No	0	Rural
17	PD	41	5	Yes	0	Rural
18	PA	39	—	—	—	Rural
19	PD	61	4	Yes	0	Rural
20	PA	—	—	—	—	Rural
21	PD	73	5	Yes	1	Rural
22	PA	70	—	—	—	Rural
23	PA	76	—	—	—	Metro
24	PD	79	6	No	0	Metro
25	PD	69	8	No	1	Metro
26	PA	76	—	—	—	Metro
27	PD	65	16	Yes	0	Metro
28	PA	60	—	—	—	Metro
29	PA	76	—	—	—	Partner
30	PA	61	—	—	—	Partner
31	PA	66	—	—	—	Partner
32	PA	58	—	—	—	Partner
33	PA	69	—	—	—	Partner
34	PA	78	—	—	—	Partner

Mean age 67 years, range 41–82 years.

Mean disease duration 10 years, range 4–21 years.

**Table 2:** Key focus group questions.

(1) Why do you walk outside your home?
(2) How is walking in the community different to walking at home?
(3) What factors make walking in the community easier?
(4) What factors make walking in the community difficult?
